# Benefits and risks of antimicrobial use in food-producing animals

**DOI:** 10.3389/fmicb.2014.00288

**Published:** 2014-06-12

**Authors:** Haihong Hao, Guyue Cheng, Zahid Iqbal, Xiaohui Ai, Hafiz I. Hussain, Lingli Huang, Menghong Dai, Yulian Wang, Zhenli Liu, Zonghui Yuan

**Affiliations:** ^1^MOA Laboratory for Risk Assessment of Quality and Safety of Livestock and Poultry Products, Huazhong Agricultural UniversityWuhan, China; ^2^Yongtgz River Fisheries Research Institute, Chinese Academy of Fishery SciencesWuhan, China; ^3^National Reference Laboratory of Veterinary Drug Residues and MOA Key Laboratory for the Detection of Veterinary Drug Residues in Foods, Huazhong Agricultural UniversityWuhan, China

**Keywords:** antimicrobial drug, benefit, risk, animal food production, public health

## Abstract

Benefits and risks of antimicrobial drugs, used in food-producing animals, continue to be complex and controversial issues. This review comprehensively presents the benefits of antimicrobials drugs regarding control of animal diseases, protection of public health, enhancement of animal production, improvement of environment, and effects of the drugs on biogas production and public health associated with antimicrobial resistance. The positive and negative impacts, due to ban issue of antimicrobial agents used in food-producing animals, are also included in the discussion. As a double-edged sword, use of these drugs in food-animals persists as a great challenge.

## INTRODUCTION

Over the decades, world population tremendously increased to 7 billion (Food and Agriculture Organization [FAO], 2012) and 870 million (12.5%) of them were estimated to be undernourished during 2010–2012 (Food and Agriculture Organization [FAO], 2012; Krehbiel, 2013). Demand for animal source food tends to be soaring day by day, especially in developing countries (Food and Agriculture Organization [FAO], 2009; Krehbiel, 2013). It is acknowledged that the antimicrobial use is one of the most successful chemotherapies. Various antimicrobials have made significant contribution for the prevention, control, and treatment of infectious diseases in animals since 1940s (Forman and Burch, 1947). Low and sub-therapeutic dose of antimicrobials plays very important role for the improvement of feed efficiency, promotion of animal growth, and prevention and control of the diseases (Dibner and Richards, 2005; Niewold, 2007). International market value of veterinary drugs (including antimicrobials) tremendously increased from $8.65 billion in 1992 to $20.1 billion in 2010 and in 2018, it is expected to increase to $42.9 billion (GIA, 2012; Reports, 2012).

It is undeniable that rational use of antimicrobials plays a vital role in the production of food animals and protecting public health, while irrational and irresponsible use may cause antimicrobial resistance. On the basis of “Swan Reports” in 1969, Great British took first action for the restriction of antibiotics, being used in animals or capable of cross-resisting with antibiotics used in human medicine. In 1973, the European Community (EC) commenced the withdrawal of some important antibiotic use as growth promoters in animal feed. After that, Sweden banned the use of all growth-promoting antibiotics in 1986. Avoparcin, bacitracin, spiramycin, tylosin, and virginiamycin were withdrawn as growth promoters in the European Union (EU) from 1995 to 1999, on the basis of precautionary principle. In 2006, all the uses of low-dose antibiotics (5~40 ppm) in food animals, including flavomycin, avilamycin, salinomycin, monensin, and other animal-specific antibiotics, were banned in the EU with the intention to avoid their negative impact of resistant development (EPC, 2005; Marshall and Levy, 2011).

The benefits and risks of antimicrobials continue to be complex and controversial issues. The risks of antimicrobial drugs to public health associated with antimicrobial resistance raised great concern recently, while the benefits of antimicrobial drugs, such as prevention and treatment of animal diseases, protection of public health, enhancement of animal production, and improvement of environment, were disregarded most of the time. Many benefit-related claims have not yet been fully demonstrated in large-scale trials, and other trials revealed that the overall impact of the short-term benefits was poorly described. This article presents the benefits and risks of antimicrobials drugs used in food animals and discusses the positive and negative effects of the ban on antimicrobial growth promoters.

## BENEFITS OF ANTIMICROBIAL DRUGS

### PREVENTION AND TREATMENT OF ANIMAL DISEASES

With intensive animal production, bacterial and parasitic diseases became more and more frequent. According to an estimate, 80 types of bacteria, such as *Escherichia coli*, *Salmonella*, and *Clostridium welchii*, posed a serious threat to poultry industry. Mastitis, caused by *Staphylococcus aureus* in dairy animals, led to a loss of $2 billion/year in the United States of America (USA) and an average cost of €485/dairy cow in the EU during 2012 (Heikkila et al., 2012). Due to infection caused by *Streptococcus pneumonia*, morbidity and mortality rates in calves increased to 40 and 20%, respectively (Akkermans and Vecht, 1994; Vogel et al., 2001). More than 50% of aquatic animals were infected by bacteria each year (Scarfe et al., 2011). *Vibrio vulnificus* became a potential health hazard for aquatic animals and human beings (Yano et al., 2004).

Approximately, 2169 parasites including 203 protozoa, 373 trematodes, 150 tapeworms, 404 nematodes, and 1030 arthropods have been found in livestock and poultry in China. About 4–20 billion dollars/year (8.3% annual output of animal husbandry) were lost due to parasitic diseases caused by coccidia, nematodes, ticks, and others in USA (Krausse and Schubert, 2010). Acute outbreaks of chicken coccidiosis paid a loss of 42 million pounds annually in the United Kingdom (Benjamin Franklyn Kaupp, 2010). In China, poultry industry had to face billions of dollars annual loss due to almost 100% chicken morbidity by coccidiosis (Zhang et al., 2013). Sheep helminthiasis led to a loss of 2.22 million dollars annually in Australia (Hosking et al., 2009; Larsen et al., 2009).

Over one hundred of antimicrobials, including β-lactams, aminoglycosides, tetracyclines, amphenicols, macrolides, sulfonamides, fluoroquinolones, lincosamides, polypeptides, and polyene, have been used in food-producing animals around the world. These antimicrobials have played an essential role in the prevention, treatment, and control of food animal diseases caused by pathogens, such as pathogenic *E. coli*, *S. aureus*, *S. pneumonia*, *Actinobacillus pleuropneumoniae*, mycoplasma, *Vibrio*, and others (Hoflack et al., 2001; Krausse and Schubert, 2010). It was reported that in USA, 52.1% of total antimicrobials were used for the treatment of infectious diseases in animals, where 90% of starter pigs, 75% of grower pigs, 50% of finisher pigs, and 25~70% of cattle received the drugs through feed (Van Lunen, 2003; USFDA, 2009; GRACE, 2013). With a dose of 40 mg/kg, avilamycin in feed could remarkably decrease the incidence of diarrhea in post-weaning pigs (Partanen et al., 2007). When salinomycin was used in sows and pigs simultaneously, incidence of diarrhea in piglets was significantly reduced and the survival rate was increased by 13.95% (Nagaraja and Taylor, 1987). Sulfonamide and folic acid supplementation in diet increased live birth rate of piglets by 1% (Lindemann and Kornegay, 1989). Hence, it is concluded that the use of antimicrobials is a primary strategy for prevention and treatment of bacterial infections in food-producing animals.

Many antimicrobials have strong activity against parasites in animals. Use of sulfonamides in animals opened a new era of anti-parasitic drugs and made lots of parasitic diseases under control. Up till now, anti-parasitic drugs have shared about one-third sale of the global veterinary drug markets. Macrolides and benzimidazoles effectively controlled nematodes. Doramectin and ivermectin helped to prevent infection of *Argulus siamensis* in carp and *Labeo rohita* (Hemaprasanth et al., 2012). In rabbit, subcutaneous injection of ivermectin, at dose of 400 mg per kg, not only helped to clinical cure ear mite infection but also prevented loss of fur and thus, played a vital role for the improvement of fur production (McKellar et al., 1992).

Conclusively, due to unique advantages, such as exact targeting of pathogens, well-known mechanisms of activity and desired stability, antimicrobials justified their usage in livestock and poultry, and played important part for prevention and treatment of bacterial and parasite diseases.

### PROTECTION OF HUMANS AGAINST ZOONOSIS

Among animal infectious and parasitic diseases, more than 200 can affect human life. *Campylobacter* spp., *Salmonella* spp., *E. coli* O157, *Vibrio parahaemolyticus*, and *Aeromonas hydrophila* from animals pose great health threat to both humans and animals (Altwegg and Geiss, 1989; Mellata, 2013). The United States Centers for Disease Control and Prevention (US-CDC) estimated that there were about 76 million annual cases of food-borne illness in USA, including 325,000 hospitalizations and 5000 deaths (Mead et al., 1999a). Annual cases of *Campylobacter* spp., *Salmonella* spp., *E. coli* O157, and *V. parahaemolyticus* were 1,963,000, 1,332,000, 62,500, and 5000, respectively (Mead et al., 1999a, b).

To some extent, antimicrobial agents guaranteed human food security and public health by controlling animal diseases and preventing transmission of zoonotic pathogens from animals to humans. When added to animal feed or drinking water, these drugs could significantly decrease the bacterial contamination in animal products. For examples, virginiamycin decreased the contamination of *Clostridium perfringens*, *Campylobacter* spp., and other food-borne pathogens in animal carcasses (Tice, 2001; Russell, 2003; Hurd et al., 2005). Salinomycin reduced infection of type C *Clostridium* in sows and weaning piglets by 43% (Nagaraja and Taylor, 1987). Neomycin in animal feed significantly reduced the number of *E. coli* O157: H7 in animal feces, and gentamycin reduced bacterial count in poultry eggs and meat (Elder et al., 2002; Doyle and Erickson, 2006). When cattle was fed with neomycin sulfate for 48 h and held for 24-h drug withdrawal period before slaughtering, it shed considerably less *E. coli* O157:H7 cells than those pen mates who did not receive the treatment (Elder et al., 2002). A farm-level study in 2008 by Ohio State University demonstrated that only 39% of hogs, raised on conventional antimicrobial operations, were infected with *Salmonella*, while those were 54% in case of antimicrobial-free operations (Nunes, 2008; AMI, 2010). Florfenicol (10 mg/kg) presented 100% efficiency for the treatment of *A. hydrophila* of *Piaractus mesopotamicus* (Carraschi et al., 2012). Oxytetracycline hydrochloride or norfloxacin in bait feed reduced the number of *A. hydrophila* in water by 46.86~66.24%, indicating that the risk of the bacterial infection to humans be decreased.

### ENHANCEMENT OF ANIMAL PRODUCTION

In 1943, a few farmers in USA found that pigs fed with penicillin-fermented mixture grew faster (Wahlstrom et al., 1950; Hewes, 1955; Taylor and Gordon, 1955). In 1946, Moore found that low dose of streptomycin stimulated chick’s growth (Moore and Evenson, 1946; Dibner and Richards, 2005). Subsequently, chlortetracycline, doxycycline, and sulfonamides helped growth promotion in calves, pigs, and chicken. Cunha from University of Florida and Stokstad from University of Washington reported that penicillin in fermentation mixture functioned as a growth promoter for food-animals (Cunha et al., 1951). Legal use of the antimicrobials in feed has a history of over 60 years. Food and Drug Administration in USA (US FDA) approved these drugs as growth promoters for animals in 1951. Till 1978, 47.9% of the antimicrobials were used to be added in animal feed in USA. About 60% of poultry, 93% of chicken, 97% of growing pigs, and 80% of fattening pigs received antimicrobials through diet during the early 1990s. More than 40% of the drugs were added in animal feed at subtherapeutic level for improving animal production in USA during 1990s (Van Lunen, 2003). With a substantial contribution to the development of food-animal production at global level, veterinary antimicrobials tend to be necessities to cope with increasing food demand for humans.

Role of antimicrobials for the improvement of feed conversion ratio (FCR), animal growth, and reproductive performance has been well proven as given in **Table 1**, and discussed under following points (Cromwell, 2002). (1) Orally administered antimicrobials in pigs increased diet digestibility and improved feed utilization efficiency by 1.7~5.1% and 6.9~7%, respectively (Cromwell, 1999; Hardy, 1999; JETACAR, 1999; Van Lunen, 2003). (2) Addition of the drugs (e.g., chlortetracycline, sulfonamide, folic acid, carbadox, tilmicosin, tylosin, or sulfamethazine) in feed could remarkably improve the conception rate, farrowing rate, milk secretion, productive efficiency of sow, and live birth rate of piglet (Soma and Speer, 1975; Lindemann and Kornegay, 1989; Alexopoulos et al., 1998; Kantas et al., 1998; Weber et al., 2001; Partanen et al., 2007). (3) Feeding antimicrobials to pigs increased their weight gain by 1.9~16.4% (Nagaraja and Taylor, 1987; Cromwell, 1999; JETACAR, 1999; Van Lunen, 2003; IFAH-EuroP, 2005). (4) Administration of antibiotics (bacitracin zinc, colistin sulfate, flavomycin, and florfenicol) in fish diet significantly improved the feed conversion and promoted their growth (He et al., 2011; Zhou et al., 2011). (5) Antimicrobial (tiamulin, nosiheptide, salinomycin, and tylosin) supplementation could also improve the carcass quality by decreasing the fat thickness and increasing the lean meat of food-producing animals (Cromwell et al., 1984a, b; Cromwell and Stahly, 1985; Lindemann et al., 1985; Van Lunen, 2003).

**Table 1:** Part of evidences for the role of antimicrobials on feed utilization, growth promotion, reproductive performance, and carcass quality.

Example no	Reference	Drugs and animals	Parameters of animal production	Increase or decrease rate
1	Hardy (1999), Van Lunen (2003)	Antimicrobials to growing and fattening pigs	Digestion of energy	5.10%↑	
			Digestion of nitrogen	1.80%↑
			Digestion of phosphorus	3.40%↑
2	Van Lunen (2003)	Antimicrobials to swine	Feed utilization	7%↑
			Average weight gain	3.3–8.8%↑
3	JETACAR (1999), Van Lunen (2003)	Antimicrobials to young pigs	Feed utilization	4.60%↑
			Average weight gain	6.80%↑
		Antimicrobials to grower pigs	Feed utilization	1.70%↑
			Average weight gain	1.90%↓
4	Cromwell (1999), Van Lunen (2003)	Antimicrobials to piglet	Feed utilization	6.90%↑
5	IFAH-EuroP (2005)	Antimicrobials to food animal	Average weight gain	4–5%↑
6	Cromwell (1999), Van Lunen (2003)	Antimicrobials to piglet	Average weight gain	16.40%↑
7	Nagaraja and Taylor (1987)	Salinomycin to weanling piglets	Average weight gain	15.82%↑
8	He et al. (2011), Zhou et al. (2011)	(Bacitracin zinc, colistin sulfate, Flavomycin and florfenicol) to (Carassius, Carp or hybrid tilapia)	Average weight gain	24.5~40.87%↑
9	Soma and Speer (1975)	Chlortetracycline to sow	Conception rate	4.10%↑
			Farrowing rate	5.80%↑
10	Lindemann and Kornegay (1989)	Folic acid to sow	Gestation gain	18%↑
11	Cromwell and Stahly (1985), Cromwell et al. (1984a, b), Lindemann et al. (1985)	Tiamulin, nosiheptide, and salinomycin to pig	Thickness of backfat	9.7%↓
			Thickness of total fat	8%↓
			Eye muscle area	9.80%↑
			Lean meat	4.40%↑

*↑ Denotes increase, while ↓ denotes decrease.*

A lot of studies were carried out to find the mechanism involved in beneficial aspects of antimicrobials in animals. Jukes, Franti, and other scientists proved that the drugs attenuated intestinal wall and improved the digestibility of nutrients (Manson, 1968; Falkow, 1970; Jukes, 1970; Franti et al., 1971, 1972; Dibner and Richards, 2005). Midtvedt (1986) and Norin (1997) confirmed that oral doses of antimicrobials improved the structure of intestinal flora (Midtvedt, 1986; Norin, 1997). Salinomycin and avilamycin in feed improved the bioavailability of α-tocopheryl acetate in broilers by altering lipid absorption (Knarreborg et al., 2004). According to a previous review by Allen et al. (2013), antimicrobials have multi-functional role in animals, elaborated under following points: (1) these could reduce the colonization of intestinal bacteria and inhibit the growth of pathogenic microorganisms; (2) by decreasing the thickness of mucous membrane, led to more absorption of nutrients and reduced fermentation; (3) they directly neutralized the host immune response. In short, antimicrobials could affect the host intestinal flora, intestinal physiology, and immune system, and consequently, prevent disease, improve feed conversion, and enhance the growth of animals (Niewold, 2007). Till now, there are no appropriate alternatives which can replace antimicrobial growth promoters, in case those remain banned. Although numerous feed additives, mainly pre- and pro-biotic products, are commercially available now and seem to have potential to replace these growth promoters, but their true efficacy and mechanism of action in domestic animals remain unclear because of some inconsistent experimental results (Gaggia et al., 2010; Allen et al., 2013). Additionally, lack of safety evaluation and poor stability also limited the practical use of pre- and pro-biotic as feed additives.

### IMPROVEMENT OF ENVIRONMENT

According to a report of Center for Food Safety (CFS, 2013) and Food and Agriculture Organization (FAO, 2006), housing stress, due to over-crowding of animals, creates sweeping and devastating impacts on the natural and human environment leading to global warming, land degradation, air and water pollution, and loss of biodiversity. Livestock waste is one of the major sources of greenhouse gases, as the abnormal fermentation of gastrointestinal tract contents can produce lots of methane, ammonia, carbon dioxide, as well as stench gases (e.g., nitrate, ethylene acid, methyl mercaptan, hydrogen sulfide, methylamine, and trimethylamine). Fecal waste of animals generally contains 24% protein and 6.1~17.96% amino acids. Nitrogen and phosphorus in the waste lead to environmental pollution, water eutrophication and ecological imbalance.

Some antimicrobials in feed could inhibit the abnormal fermentation and consequently, reduce the emission of greenhouse gases (mainly CH_4_). For example, ionophores (monensin, lasalocid, and salinomycin), amoxicillin, ovoparcin, nigericin, or laidlomycin inhibited rumen microbial fermentation at different levels and thus, reduced the proportion of volatile fatty acids (VFA) and methane (Van Nevel and Demeyer, 1995; Fellner et al., 1997; Domescik and Martin, 1999). Since the mid-1970s, ionophorous antibiotics have been widely used as feed additives in ruminants due to their favorable effects on rumen fermentation and methane reduction (Kobayashi, 2010). Due to the efficacy and affordable price, ionophores have widely been used to reduce methane emission from livestock (Hook et al., 2010; Kobayashi, 2010). When ionophores (monensin and lasalocid) were mixed with rumen microorganisms *in vitro*, these inhibited methane by 50 and 44%, respectively and decreased NH_3_ by more than 50% (Russell and Jeraci, 1984). In rumen models, ionophores (monensin, lasalocid, and salinomycin) inhibited 10~20% lipolysis and biological hydrogenation (Van Nevel and Demeyer, 1977, 1995). The effects of the antibiotics on the abatement of methane production may be attributed to a selective antimicrobial action on rumen microbes (protozoa, ruminococci, streptococci, and lactobacilli). Addition of monensin and lasalocid in cow forage killed 82.5 and 76.8%, respectively, of the intestinal ciliated protozoa in rumen and hence, reduced the production of methane and VFAs (acetic acid and propionic acid) by ciliates (Guan et al., 2006). Generally, the gas production was reduced from 4 to 31% by monensin (Schelling, 1984; Rumpler et al., 1986; Kobayashi, 2010). A recent report has indicated that long-term administration of monensin in dairy cattle steadily reduced methane by 7% and this reduction persisted for 6 months with no adverse effect on milk yield (Odongo et al., 2007). However, previous studies also found that both, the methane level and protozoal number, returned to baseline after long-term administration of high concentration of the antibiotic (Guan et al., 2006; Odongo et al., 2007; Hook et al., 2010). The efficiency of monensin supplementation, for reducing methane output in ruminants, appeared to be different in the degree of abatement depending on the diet and animal used (Guan et al., 2006; Odongo et al., 2007; Hook et al., 2010). Effect of the drug on the methane levels in rumen was closely related to the ciliated, protozoal population. Microbial consortia, like protozoal population, in the ruminant gut may adapt to the antibiotics leading to the recovery of methane production yield, in case of a long-term usage. Therefore, the ciliates in the rumen may impact the outcome of antimicrobial supplementation, with adaptation being a possibility (Guan et al., 2006; Hook et al., 2010).

Through manure application, antibiotics got released into soil and could be absorbed by plants in arable land. Certain species of plants have the ability to bio-accumulate sulfamethoxine in their roots and stems, and this bioaccumulation was often higher in roots than in stems (Sarmah et al., 2006). Low concentrations of chlortetracycline and oxytetracycline in the soil media could markedly affect plant growth and development (Sarmah et al., 2006). However, there was a large variation in sensitivity among plant species to the soil used as the growth media (Sarmah et al., 2006).

When residues seeped into water, certain antimicrobials also played significant role in the prevention of water eutrophication for aquatic animals. For example, chlortetracycline, lomefloxacin hydrochloride, and sulfamethoxazole strengthened the absorption of nitrogen and phosphorus in water by aquatic plants, and chlortetracycline effectively removed 25% of water nitrate and nitrogen (Dodds et al., 1991; Jones, 2010). On the other hand, the presence of antibiotic residues in environment may cause some adverse impacts, like acute and chronic toxicity, during early life stages of different aquatic organisms (Halling-Sorensen et al., 2003; Sarmah et al., 2006).

### NEGATIVE IMPACT OF ANTIMICROBIAL BAN

Ban on addition of sub-therapeutic antimicrobials in feed appeared to result in a certain extent of recovery of some bacteria and some unintended impacts on animal health and welfare (Drouin, 1999; Rose et al., 1999; Lovland and Kaldhusdal, 2001; Jensen et al., 2003). Emergence of *E. coli* and *Lawsonia intracellularis* infection in post-weaning pigs was significantly increased and consequently, the morbidity and mortality due to diarrhea were considerably increased (Casewell et al., 2003; Dibner and Richards, 2005). In Denmark, the mortality rate in weaning piglets increased from 2.7% (before the ban) to 3.5% (after the ban) and the morbidity rate of enteric infections in post-weaning pigs increased by 600% (Casewell et al., 2003; Dibner and Richards, 2005). In Sweden, chronic infections due to *E. coli* and *L. intracellularis* became more common and the mortality in weaning pigs increased by 1.5% (Wierup, 2001; Casewell et al., 2003).

**FIGURE 1:**
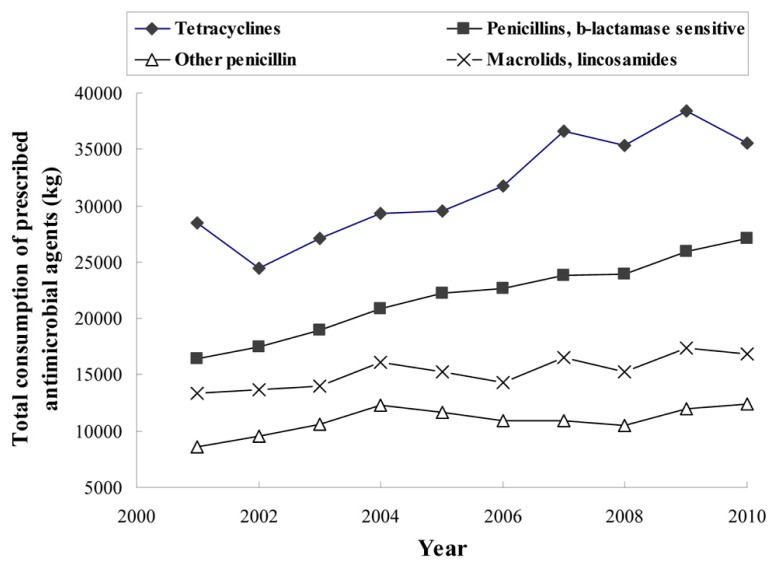
**Estimated total consumption (kg) of prescribed antimicrobial agents for production animals in Denmark (data obtained from** DANMAP, 2010).

To control animal diseases and to keep animals healthy, more therapeutic antimicrobials had to be used after the ban. It was reported that the usage amount of therapeutic antimicrobials in Denmark increased by 33.6%, from 48 tons/year in 2001 to 125.5 tons/year in 2010 (DANMAP, 2010). The increased amount of therapeutic antimicrobials was equal to or even more than the total quantity of antimicrobials being used before the ban (Phillips, 1999; Casewell et al., 2003; Phillips et al., 2004a, b; Turnidge, 2004; DANMAP, 2010). As shown in **Figure 1**, therapeutic use of tetracycline, penicillins, and macrolides markedly increased from 28.5, 16.4, and 13.4 tons in 2001 to 35.55, 27.1, and 16.8 tons in 2010, respectively (Phillips, 2007; DANMAP, 2010).

Withdrawal of low-dose antimicrobial use as feed additives may increase the level of pathogens such as *Salmonella* spp., *Campylobacter* spp., *Clostridium* and *E. coli* O157 in animal gut, boost the contamination of food and environment, and hence, enhance the opportunities for humans to be infected by these pathogens. Population of *Campylobacter* in broilers fed without antimicrobials was threefold higher than that in the broilers fed with any antimicrobials (Heuer et al., 2001). Incidence of food-borne *Campylobacter* in EU has been increased after the ban issue. Recovery of *Clostridium* from animal meat was also significantly increased in EU after its ban policy (Jones, 2000; Poduval et al., 2000). As a consequence, clostridial infections resulted in an outbreak affecting large human population in Demark and pointed out the high level of threat to public health (DANMAP, 2010). It is known that clostridial necrotic enteritis in animals is suppressed by some of the banned antimicrobials (e.g., virginiamycin). In the absence of these antibiotics, the bacterial population may increase in animal guts and colonization may lead to poor weight uniformity and fragile intestines in pigs and chicks. During food processing, infected animal carcasses could be the sources of contamination for food-borne pathogens and thereby, jeopardize food hygiene (Tice, 2001; Russell, 2003). Bacterial contamination of meat may, therefore, increase the risk of human infections. Contrary to EU, incidence of food-borne diseases in USA was declined by 23% in 1996 and among those, the infections due to *Campylobacter* and *Salmonella* were decreased by 30 and 17%, respectively. It was believed that ban of virginiamycin in USA might annually contribute to the death of 40,000 people, infected by *Campylobacter* (Cox, 2005).

The ban may lead to increased food-borne infections and elevated usage of therapeutic antimicrobials in both animals and humans. It is noteworthy that therapeutic use of antimicrobial agents in animals has a close relationship with the drugs used in humans with respect to the types of drugs used (Cook, 1999). The increased therapeutic use in animals may contribute to a worse, drug-resistance scenario both in animals and humans. It was noted that clinical isolates of vancomycin- or teicoplanin-resistant *Enterococci* from humans were very uncommon and the cases of quinupristin/dalfopristin-resistant *Enterococcus faecium* were very rare before the ban. Similarly, resistant *E. faecium* burden markedly increased and became a big challenge after the ban (Phillips, 2007).

**FIGURE 2:**
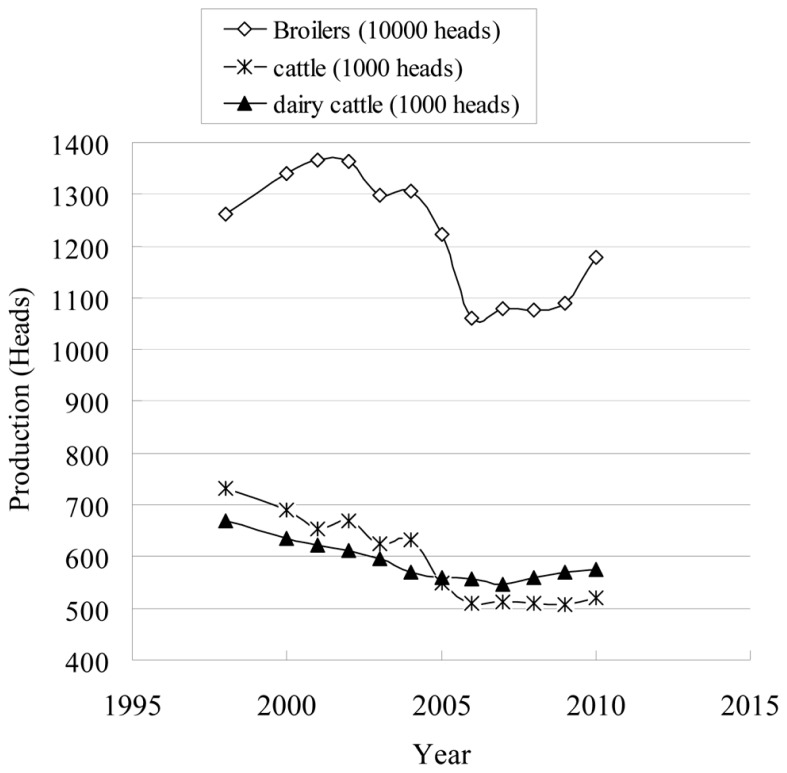
**Production of food animals in Denmark from 1998 to 2010 (data obtained from** DANMAP, 2010).

After the European ban on growth-promoting antibiotics, it was found that FCR (total kg of feed used per grow out/total kg of live weight per grow out) in broiler was decreased by 0.016 kg/kg from November 1995 to May 1999 (1.78–1.796) in Denmark. Feed efficiency raised to a higher value of 1.83 immediately after the restrictions and more than 1.84 in late 1999 (Emborg et al., 2002; Dibner and Richards, 2005). Average daily gain of weaning piglets in Denmark was decreased from 422 g in 1995 to 415 g in 2001 (Casewell et al., 2003; Dibner and Richards, 2005). Production of broilers, cattle, and dairy cows was significantly decreased in 2006, as shown in **Figure 2**. In Sweden, weight gain of post-weaning piglets was reduced and feed costs were significantly increased after the abolishment of growth-promoting antimicrobial agents (Wierup, 2001; Casewell et al., 2003). Even after 10 years, aquaculture production in Sweden was unable to return to the past level (Casewell et al., 2003).

If the use of antimicrobials is banned in USA, FCR may decline by 5% and more than 1100 km^2^ area is required to plant corn and soybeans to meet the demand of feedstuff production. Consequently, required facilities for livestock and poultry production will be correspondingly increased by 100 million m^2^, and farming area will also be increased by 500 million m^2^ to obtain the current level of animal production (Casewell et al., 2003). It was estimated that withdrawal of antimicrobial growth promoters might lead to a loss of 5~10 or even 40 dollars/pig in USA (Matthews, 2001). It does not seem to be a high cost for the developed countries but what will be the consequences if these antimicrobials are banned worldwide? It was also investigated that 25% of the current poultry industry and $3 billions would be additionally required to attain the current annual animal production (Rodehutscord et al., 2002; IFAH-EuroP, 2005). In conclusion, keeping in view the ever-increasing world population and its demands, more food animals should be raised to meet the food supply demand in the case of growth-promoting antibiotics remain prohibited and this increased number of animals will again lead to the increase in greenhouse gas emission and deeper environmental pollution.

## RISKS OF ANTIMICROBIALS

### INHIBITION OF BIOGAS PRODUCTION

Following the wide use of antimicrobial drugs in intensive animal production for growth promotion and prevention or treatment of disease, a large proportion of ingested drugs are excreted in manure and end up with livestock wastewater. Excreted antibiotics in the environment may partially inhibit methanogenesis in anaerobic waste-storage facilities, commonly used at Concentrated Animal Feeding Operation (CAFOs), and thus, decrease the rate at which bacteria metabolize animal waste products (Loftin et al., 2005; Sarmah et al., 2006).

During the anaerobic digestion of livestock waste, certain antimicrobials, including amoxicillin, aureomycin, oxytetracycline, thiamphenicol, florfenicol, sulfadimethoxine, and tylosin, had inhibitory effects on methane production (Lallai et al., 2002; Sun et al., 2009; Shi et al., 2011; Amin et al., 2012). However, no inhibitory effect but a stimulus for methane production was observed during anaerobic digestion of piggery wastewater in the presence of 10 mg/L florfenicol, amoxicillin, aureomycin, and sulfadimethoxine, while only the combination of high concentration of certain antimicrobials (130 mg/L florfenicol, 210mg/L amoxicillin, 10mg/L doxycycline, and 210 mg/L sulfadimethoxine) could decrease the methane production rate (Sun et al., 2009). Biogas volume, produced from per unit weight of biomass, was decreased with increasing concentrations of antibiotics, such as oxytetracycline, amoxicillin, and tylosin, and the inhibitory concentrations of oxytetracycline, amoxicillin, and tylosin were 8000, 9000, and 9000 mg/L, respectively (Amin et al., 2012). Only high concentration of thiamphenicol (160 mg/L), amoxicillin (120 mg/L), tetracycline (50 mg/L), and sulfamethoxydiazine (50 mg/L) had inhibitory effect on biogas production in the anaerobic digestion of pig waste slurry (Lallai et al., 2002; Shi et al., 2011). Actually, it is too difficult to attain those high concentrations of antibiotics in the excreta.

### ANTIMICROBIAL RESISTANCE CONCERNS

Misuse and overuse of antimicrobial may culminate in the development of drug-resistant pathogens resulting in poor response to treatment. Long-term and low-level exposure to antimicrobials may have greater selective potential than short-term and full-dose therapeutic use. A study observed that the percentage of tetracycline resistance genes in the fecal flora of conventionally raised feedlot steers was significantly higher than that in fecal samples from antimicrobial-free cattle (Harvey et al., 2009). Additionally, use of single antimicrobial may induce cross-resistance to antimicrobials used for animal and human medical therapy. For example, chlortetracycline use in growth rations was associated with ampicillin and tetracycline resistance in generic fecal *E. coli*, isolated from swine farms (Varga et al., 2009). Addition of chlortetracycline and sulfamethazine in cattle feed may be associated with higher prevalence (three to four fold greater than the control) of ampicillin- and tetracycline-resistant *E. coli*, isolated from the feces of treated animals (Alexander et al., 2010). Therefore, how to use antimicrobials, for effective treatment of bacterial and parasitic infections in food-producing animals, became the most important question for their use by avoiding the resistance development.

Regarding public health risk, more concern has been raised for the use of antibiotics in animals that may represent a potential threat to human health because the resistant pathogens in animals may transmit to humans and cause treatment failure of human medicines. A longitudinal study of the relationship between antimicrobial-resistant *E. coli* from human wastewater and swine fecal samples reported that the use of injectable (e.g., ceftiofur sodium) or oral (e.g., chlortetracycline) antibiotics may contribute to the high levels of *E. coli* resistance in swine and human isolates. Thus, slaughter plant workers may be at higher risk of carrying multidrug-resistant *E. coli* as compared to workers dealing with other animals (Alali et al., 2008). A study analyzed the correlation between *E. coli* isolates from human blood stream and food-producing animals (poultry, pigs, and cattle) for the prevalence of antimicrobial resistance in 11 countries during 2005–2008. Results revealed that there were strong and significant correlations between the strains from animals (especially poultry and pigs) and humans for resistance to multiple drugs (especially ampicillin, aminoglycoside, and fluoroquinolone; Vieira et al., 2011). However, there is not enough and direct evidence to support the hypothesis that a large proportion of resistant isolates in humans be derived from animal source foods. Some recent studies suggested that some resistant isolates from humans were more easily transmitted from companion animals (having close contact) or birds (by bird droppings especially during their migration; Haenni et al., 2012; Loncaric et al., 2013). The resistant bacteria may also be released into the environment by humans and then transferred into new hosts in the environment (Hower et al., 2013; Verkade and Kluytmans, 2013). Based on the results of some current studies, Dr. Hurt pointed out that public health risk due to infected animals need more attention than antibiotic resistance concern (Hurd et al., 2008, 2012). Therefore, further efforts are required for the risk assessment of antimicrobial use in food animals to check their potential impact on public health.

### POSITIVE EFFECTS OF ANTIMICROBIAL BAN

Aim for the withdrawal of antimicrobial agents used was to prevent humans and animals from drug resistance. The ban on antimicrobial growth promoters led to decreased drug resistance in some bacteria. For example, according to report from the DANMAP, substantial reductions (from 80 to 2%) in the prevalence of vancomycin-resistant enterococci (VRE) were observed after ban of avoparcin as the growth promoter during 1995 and 2010 (DANMAP, 2010). *E. faecium* and *Enterococcus faecalis* are two of the most common *Enterococci* species and in Europe, only vancomycin-resistant *E. faecium* (VREF) is highly prevalent in poultry (Werner et al., 2008). VREF was still present as a threat in the food chain even after 15 years of the EU ban on avoparcin and could be detected in 47% of the broiler feces with a selective enrichment method (Garcia-Migura et al., 2007; DANMAP, 2010).

The ban on tylosin as the growth promoter had a remarkable effect on the level of macrolide (erythromycin) resistance in *Campylobacter coli* (most common *Campylobacter* species in pigs) from pigs as it decreased from 66 to 20% in Denmark between 1998 and 2005 (Hammerum et al., 2007). However, DANMAP data showed that during 2006–2010, macrolide resistance in *C. coli* varied within the range of 10–20% without significant reduction (DANMAP, 2010). In contrast to EU, macrolides (e.g., tylosin) in USA had been approved for usage in food-producing animals as growth promoter for decades. Macrolide resistance in *C. coli* isolated from poultry, although higher than that in *C. jejuni*, has no significant change (5–20%) between 2002 and 2010.

Use of enrofloxacin in poultry was withdrawn by US-FDA in 2005 because it was supposed to induce fluoroquinolone resistance in *Campylobacter* and *Salmonella* from poultry and contribute to the antibiotic treatment failure in humans [Food and Drug Administration (FDA), 1998, 2002; Nelson et al., 2007]. After withdrawal of enrofloxacin from poultry, the rate of fluoroquinolone resistance in *Campylobacter* and *Salmonella* had been reduced in chicken during 2005–2007 (NARMS, 2010). Human clinicians also observed a reduction in domestically acquired *Campylobacter* and *Salmonella* infections with decreased susceptibility to fluoroquinolones, and it was thought to be a great achievement regarding public health (Nelson et al., 2007). However, the incidence rate of fluoroquinolone-resistant *C. jejuni*, from chicken breast, again increased (15.2~22.5%) in 2008–2010. Similarly, fluoroquinolone-resistant *Campylobacter* from broilers was also raised from 5.3 to 8.6% and from 0.8 to 7.7% in Denmark and Japan, respectively (CIPARS, 2008; JVARM, 2008; DANMAP, 2010; NARMS, 2010).

### CONCLUSION AND PERSPECTIVES

Definite targeting of pathogens, well-known mechanisms of activity, and preferable stability for administration are the unique advantages of antimicrobial use in food-animals for the prevention and treatment of bacterial and parasitic diseases, improvement of animal production performance, and protection of environment and public health. Withdrawal of antimicrobial use from food-producing animals may bring adverse effects on the production of food derived from animals and thus, on public health. EU banned only low-dose antibiotics (5~40 ppm) for their use as growth promoters in food animals. Until now, without helping for the cause, it led to some negative effects on food animal production and public health. What will happen if the antimicrobial agents are banned worldwide, especially in some developing countries with rapid increase in human population and food demand?

As a double-edged sword, non-rational uses of veterinary antimicrobials may result in pressure selection of antimicrobial resistant pathogens which may endanger both the animal and public health. Additionally, the presence of antibiotic residues in the environment, associated with overuse of antimicrobial drugs, may adversely influence the manure treatment systems by inhibition of biogas production. An economic analysis about use and withdrawal of antimicrobial growth promoters in USA revealed that the withdrawal may cause increased cost ($10/person) for food consumption and antibiotic-resistant infections cost the US healthcare system an excess of $20 billion ($60/person) annually (APUA, 2010). However, it is unknown that how much of these $20 billion is due to antimicrobial resistance associated with their use in food-producing animals.

Recently, the US FDA has also proposed restrictions on the antimicrobial growth promoters because some available information and evidences suggested that the sub-therapeutic use may increase the risk of antimicrobial resistance. To make wise strategies for controlling antimicrobial resistance and effectively respond to the public health concerns associated with drug resistance, FDA believes that it is very important and imperative to consider how antimicrobial drugs are being used and how to address their injudicious uses in nature. Only rational use and effective regulation can ensure a benefit–risk balance of the antimicrobial application in animal production. However, long-term policies will be required for the international regulation of antibiotic use in food-producing animals.

## Conflict of Interest Statement

The authors declare that the research was conducted in the absence of any commercial or financial relationships that could be construed as a potential conflict of interest.

## References

[B1] Akkermans J. P. Vecht U. (1994). Streptococcal infections as cause of death in pigs brought in for necropsy. *Tijdschr. Diergeneeskd.* 119 123–128 8134911

[B2] Alali W. Q. Scott H. M. Harvey R. B. Norby B. Lawhorn D. B. Pillai S. D. (2008). Longitudinal study of antimicrobial resistance among *Escherichia coli* isolates from integrated multisite cohorts of humans and swine. *Appl. Environ. Microbiol.* 74 3672–3681 10.1128/AEM.02624-07 18424541 PMC2446572

[B3] Alexander T. W. Inglis G. D. Yanke L. J. Topp E. Read R. R. Reuter T. (2010). Farm-to-fork characterization of *Escherichia coli* associated with feedlot cattle with a known history of antimicrobial use. *Int. J. Food Microbiol.* 137 40–48 10.1016/j.ijfoodmicro.2009.11.008 19963297

[B4] Alexopoulos C. Tsinas A. Kantas D. Florou-Paneri P. Read M. P. Vassilopoulos V. (1998). A dose titration study on the effect of virginiamycin on specific blood parameters and milk quality in the sow. *Zentralbl. Veterinarmed.* 45 535–542 10.1111/j.1439-0442.1998.tb00857.x 9857831

[B5] Allen H. K. Levine U. Y. Looft T. Bandrick M. Casey T. A. (2013). Treatment, promotion, commotion: antibiotic alternatives in food-producing animals. *Trends Microbiol.* 21 114–119 10.1016/j.tim.2012.11.001 23473629

[B6] Altwegg M. Geiss H. K. (1989). Aeromonas as a human pathogen. *Crit. Rev. Microbiol.* 16 253–286 10.3109/10408418909105478 2649316

[B7] AMI. (2010). *Antibiotic Use in Livestock Production: Ensuring Meat Safety* American Meat Institute. Available at: http://www.MeatAMI.com

[B8] Amin M. M. Hashemi H. Ebrahimi A. Ebrahimi A. (2012). Effects of oxytetracycline, tylosin, and amoxicillin antibiotics on specific methanogenic activity of anaerobic biomass. *Int. J. Environ. Health Eng.* 1 1–4 10.4103/2277-9183.102356

[B9] APUA. (2010). *The Cost of Antibiotic Resistance to U.S Families and the Health Care System*. A.f.t.P.U.o. ed Available at: http://www.tufts.edu/med/apua/consumers/personal_home_5_1451036133.pdf

[B11] Carraschi S. P. da Cruz C. Machado Neto J. G. Ignácio N. F. Barbuio R. Machado M. R. (2012). Histopathological biomarkers in pacu (*Piaractus mesopotamicus*) infected with *Aeromonas hydrophila* and treated with antibiotics. *Ecotoxicol. Environ. Saf.* 83 115–120 10.1016/j.ecoenv.2012.06.016 22766414

[B12] Casewell M. Friis C. Marco E. McMullin P. Phillips I. (2003). The European ban on growth-promoting antibiotics and emerging consequences for human and animal health. *J. Antimicrob. Chemother.* 52 159–161 10.1093/jac/dkg313 12837737

[B13] Center for Food Safety [CFS]. (2013). *Animal Factories and Environmental Pollution*. Available at:http://www.centerforfoodsafety.org/issues/307/animal-factories/animal-factories-and-environmental-pollution#

[B14] CIPARS. (2008). *Canadian Integrated Program for Antimicrobial Resistance Surveillance Annual Report 2008*. Available at: http://www.phac-aspc.gc.ca/cipars-picra/2008/index-eng.php

[B15] Cook R. (1999). EU ban on four antibiotic growth promoters. *Vet. Rec*. 144 158 10074667

[B16] Cox L. A. Jr. (2005). Potential human health benefits of antibiotics used in food animals: a case study of virginiamycin. *Environ. Int.* 31 549–563 10.1016/j.envint.2004.10.012 15871160

[B17] Cromwell C. L. (2002). Why and how antibiotics are used in swine production. *Anim. Biotechnol.* 13 7–27 10.1081/ABIO-120005767 12212945

[B18] Cromwell G. L. (1999). “Subtherapeutic use of antibiotics for swine: performance, reproductive efficiency and safety issues,” in *Proceeding of 40th George A. Young Swine Health and Management Conference* Lincoln 69–87

[B19] Cromwell G. L. Stahly T. S. (1985). Efficacy of tiamulin as a growth promotant for growing swine. *J. Anim. Sci.* 60 14–19 3972735 10.2527/jas1985.60114x

[B20] Cromwell G. L. Stahly T. S. Jensen A. H. Plumlee M. P. Krider J. L. Russett J. C. (1984a). Efficacy of thiopeptin as a growth promotant for growing barrows and gilts – a cooperative study. *J. Anim. Sci.* 59 892–895 6511682 10.2527/jas1984.594892x

[B21] Cromwell G. L. Stahly T. S. Speer V. C O’Kelly R. (1984b). Efficacy of nosiheptide as a growth promotant for growing-finishing swine – a cooperative study. *J. Anim. Sci.* 59 1125–1128 6511687 10.2527/jas1984.5951125x

[B22] Cunha T. J. Meadows G. B. Edwards H. M. Sewell R. F. Pearson A. M. Glasscock R. S. (1951). A comparison of aureomycin, streptomycin, penicillin and an aureomycin-B12 feed supplement for the pig. *Arch. Biochem.* 30 269–271 14811496

[B23] DANMAP. (2010). *DANMAP – Use of Antimicrobial Agents and Occurrence of Antimicrobial Resistance in Bacteria from Food Animals, Food and Humans in Denmark*. Available at: http://www.danmap.org

[B24] Dibner J. J. Richards J. D. (2005). Antibiotic growth promoters in agriculture: history and mode of action. *Poult. Sci.* 84 634–643 10.1093/ps/84.4.634 15844822

[B25] Dodds W. K. Priscu J. C. Ellis B. K. (1991). Seasonal uptake and regeneration of inorganic nitrogen and phosphorus in a large oligotrophic lake: size-fractionation and antibiotic treatment. *J. Plankton Res.* 13 1339–1358 10.1093/plankt/13.6.1339

[B26] Domescik E. J. Martin S. A. (1999). Effects of laidlomycin propionate and monensin on the *in vitro* mixed ruminal microorganism fermentation. *J. Anim. Sci.* 77 2305–2312 10462011 10.2527/1999.7782305x

[B27] Doyle M. P. Erickson M. C. (2006). Reducing the carriage of foodborne pathogens in livestock and poultry. *Poult. Sci.* 85 960–973 10.1093/ps/85.6.960 16776463

[B28] Drouin E. (1999). *Helicobacter pylori*: novel therapies. *Can. J. Gastroenterol.* 13 581–583 10519955 10.1155/1999/485237

[B29] Elder R. O. Keen J. E. Wittum T. E. Callaway T. R. Edrington T. S. Anderson R. C. (2002). “Intervention to reduce fecal shedding of enterohemorrhagic *Escherichia coli* O157:H7 in naturally infected cattle using neomycin sulfate,” in *American Society of Animal Science/American Dairy Science Association Joint Meeting* Quebec, 602

[B30] Emborg H. D. Ersboll A. K. Heuer O. E. Wegener H. C. (2002). “Effects of termination of antimicrobial growth promoter use for broiler health and productivity,” in *International Invitational Symposium; Beyond Anti Microbial Growth Promoters in Food Animal Production, November 6–7 2002,* Foulum 38–42

[B31] EPC. (2005). *Ban on Antibiotics as Growth Promoters in Animal Feed Enters into Effect, European Commission – IP/05/1687*. Available at: http://europa.eu/rapid/press-release_IP-05-1687_en.htm

[B32] Falkow S. (1970). Antibiotics in animal feeds. *N. Engl. J. Med.* 282 693–694 10.1056/NEJM197003192821230% 5437535

[B33] Food and Agriculture Organization (FAO). (2006). *Livestock Impacts on the Environment, Food and Agriculture Organization of the United Nations, Agriculture and Consumer Protection Department*. Available at: http://www.fao.org/ag/magazine/0612sp1.htm

[B34] Food and Agriculture Organization [FAO]. (2009). *The State of Food and Agriculture: Livestock in the Balance*. Available at: http://www.fao.org/docrep/012/i0680e/i0680e00.htm

[B35] Food and Agriculture Organization [FAO]. (2012). *The State of Food Insecurity in the World 2012.* Available at: http://www.fao.org/docrep/016/i3027e/i3027e00.htm

[B36] Food and Drug Administration (FDA). (1998). Freedom of information summary NADA 1412068, htm h.w.f.g.c.f. ed

[B37] Food and Drug Administration (FDA). (2002). *Final decision of the Commissioner Docket No. 2000N21571 with drawal of approval of the new animal drug application for enrofloxacin in poultry [EB/OL]*. Available at: [http://www.fda.gov/oc/antimicrobial/baytri.html]

[B38] Fellner V. Sauer F. D. Kramer J. K. (1997). Effect of nigericin, monensin, and tetronasin on biohydrogenation in continuous flow-through ruminal fermenters. *J. Dairy Sci.* 80 921–928 10.3168/jds.S0022-0302(97)76015-6 9178132

[B39] Forman C. R. Burch J. E. (1947). Use of sodium sulfonamides as single injection specific treatment in foot rot. *J. Am. Vet. Med. Assoc.* 111 208–214 20256929

[B40] Franti C. E. Adler H. E. Julian L. M. (1971). Antibiotic growth promotion: effects of bacitracin and oxytetracycline on intestines and selected lymphoid tissues of New Hampshire cockerels. *Poult. Sci.* 50 94–99 10.3382/ps.0500094 5550482

[B10] Franklyn B. K. (2010). *Poultry Culture, Sanitation and Hygiene* (1915). Whitefish, MT: Kessinger Publishing

[B41] Franti C. E. Julian L. M. Adler H. E. Wiggins A. D. (1972). Antibiotic growth promotion: effects of zinc bacitracin and oxytetracycline on the digestive, circulatory, and excretory systems of New Hampshire cockerels. *Poult. Sci.* 51 1137–1145 10.3382/ps.0511137 4647576

[B42] Gaggia F. Mattarelli P. Biavati B. (2010). Probiotics and prebiotics in animal feeding for safe food production. *Int. J. Food Microbiol.* 141(Suppl. 1) S15–S28 10.1016/j.ijfoodmicro.2010.02.031 20382438

[B43] Garcia-Migura L. Liebana E. Jensen L. B. Barnes S. Pleydell E. (2007). A longitudinal study to assess the persistence of vancomycin-resistant *Enterococcus faecium* (VREF) on an intensive broiler farm in the United Kingdom. *FEMS Microbiol. Lett.* 275 319–325 10.1111/j.1574-6968.2007.00911.x 17825067

[B44] GIA. (2012). *Animal Health Market to Hit $43 Billion in Five Years*. Available athttp://westernfarmpress.com/management/animal-health-market-hit-43-billion-five-years

[B45] GRACE. (2013). *The Issue: Antibiotics and the Food Animal Industry*. Available at: http://www.sustainabletable.org/257/antibiotics, GRACE Communications Foundation

[B46] Guan H. Wittenberg K. M. Ominski K. H. Krause D. O. (2006). Efficacy of ionophores in cattle diets for mitigation of enteric methane. *J. Anim. Sci.* 84 1896–1906 10.2527/jas.2005-652 16775074

[B47] Haenni M. Saras E. Chatre P. Medaille C. Bes M. Madec J. Y. (2012). A USA300 variant and other human-related methicillin-resistant *Staphylococcus aureus* strains infecting cats and dogs in France. *J. Antimicrob. Chemother.* 67 326–329 10.1093/jac/dkr499 22146878

[B48] Halling-Sorensen B. Sengelov G. Ingerslev F. Jensen L. B. (2003). Reduced antimicrobial potencies of oxytetracycline, tylosin, sulfadiazin, streptomycin, ciprofloxacin, and olaquindox due to environmental processes. *Arch. Environ. Contam. Toxicol.* 44 7–16 10.1007/s00244-002-1234-z 12434214

[B49] Hammerum A. M. Heuer O. E. Lester C. H. Agerso Y. Seyfarth A. M. Emborg H. D. (2007). Comment on: withdrawal of growth-promoting antibiotics in Europe and its effects in relation to human health. *Int. J. Antimicrob. Agents* 30 466–468 10.1016/j.ijantimicag.2007.07.012 17884357

[B50] Hardy B. (1999). “A world without growth promoters,” in *First Annual Turtle Lake Science Conference* (Nottingham: Nottingham University Press).

[B51] Harvey R. Funk J. Wittum T. E. Hoet A. E. (2009). A metagenomic approach for determining prevalence of tetracycline resistance genes in the fecal flora of conventionally raised feedlot steers and feedlot steers raised without antimicrobials. *Am. J. Vet. Res.* 70 198–202 10.2460/ajvr.70.2.198 19231951

[B52] He S. Zhou Z. Meng K. Zhao H. Yao B. Ringo E. (2011). Effects of dietary antibiotic growth promoter and *Saccharomyces cerevisiae* fermentation product on production, intestinal bacterial community, and nonspecific immunity of hybrid tilapia (*Oreochromis niloticus* female × *Oreochromis aureus* male). *J. Anim. Sci.* 89 84–92 10.2527/jas.2010-3032 20852079

[B53] Heikkila A. M. Nousiainen J. I. Pyorala S. (2012). Costs of clinical mastitis with special reference to premature culling. *J. Dairy Sci.* 95 139–150 10.3168/jds.2011-4321 22192193

[B54] Hemaprasanth K. P. Kar B. Garnayak S. K. Mohanty J. Jena J. K. Sahoo P. K. (2012). Efficacy of two avermectins, doramectin and ivermectin against *Argulus siamensis* infestation in Indian major carp, *Labeo rohita*. *Vet. Parasitol.* 190 297–304 10.1016/j.vetpar.2012.05.010 22673107

[B55] Heuer O. E. Pedersen K. Andersen J. S. Madsen M. (2001). Prevalence and antimicrobial susceptibility of thermophilic *Campylobacter* in organic and conventional broiler flocks. *Lett. Appl. Microbiol.* 33 269–274 10.1046/j.1472-765X.2001.00994.x 11559399

[B56] Hewes C. G. (1955). The influence on the growth and progeny of the guinea pig resulting from oral administration of aureomycin (chloretracycline) and penicillin. *J. Nutr.* 57 353–360 13272077 10.1093/jn/57.3.353

[B57] Hoflack G. Maes D. Mateusen B. Verdonck M de Kruif A. (2001). Efficacy of tilmicosin phosphate (*Pulmotil premix*) in feed for the treatment of a clinical outbreak of *Actinobacillus pleuropneumoniae* infection in growing-finishing pigs. *J. Vet. Med.* 48 655–664 10.1046/j.1439-0450.2001.00492.x 11765801

[B58] Hook S. E. Wright A. D. McBride B. W. (2010). Methanogens: methane producers of the rumen and mitigation strategies. *Archaea* 2010 945785 10.1155/2010/945785 PMC3021854 21253540

[B59] Hosking B. C. Griffiths T. M. Woodgate R. G. Besier R. B. Le Feuvre A. S. Nilon P. (2009). Clinical field study to evaluate the efficacy and safety of the amino-acetonitrile derivative, monepantel, compared with registered anthelmintics against gastrointestinal nematodes of sheep in Australia. *Aust. Vet. J.* 87 455–462 10.1111/j.1751-0813.2009.00511.x 19857240

[B60] Hower S. Phillips M. C. Brodsky M. Dameron A. Tamargo M. A. Salazar N. C. (2013). Clonally related methicillin-resistant *Staphylococcus aureus* isolated from short-finned pilot whales (*Globicephala macrorhynchus*), human volunteers, and a Bayfront Cetacean Rehabilitation Facility. *Microb. Ecol.* 65 1024–1038 10.1007/s00248-013-0178-3 23508733

[B61] Hurd H. S. Brudvig J. Dickson J. Mirceta J. Polovinski M. Matthews N. (2008). Swine health impact on carcass contamination and human foodborne risk. *Public Health Rep*. 123 343–351 19006976 10.1177/003335490812300314 PMC2289987

[B62] Hurd H. S. Gailey J. K. McKean J. D. Griffith R. W. (2005). Variable abattoir conditions affect *Salmonella enterica* prevalence and meat quality in swine and pork. *Foodborne Pathog. Dis.* 2 77–81 10.1089/fpd.2005.2.77 15992301

[B63] Hurd H. S. Yaeger M. J. Brudvig J. M. Taylor D. D. Wang B. (2012). Lesion severity at processing as a predictor of *Salmonella* contamination of swine carcasses. *Am. J. Vet. Res.* 73 91–97 10.2460/ajvr.73.1.91 22204293

[B64] IFAH-EuroP. (2005). *IFAH – Europe Annual Report 2005*. Available at: http://www.ifaheuroPe.org/Publieations/IFAH_EuroPe_AR_2005.Pdf

[B65] Jensen G. B. Hansen B. M. Eilenberg J. Mahillon J. (2003). The hidden lifestyles of *Bacillus cereus* and relatives. *Environ. Microbiol.* 5 631–640 10.1046/j.1462-2920.2003.00461.x 12871230

[B66] JETACAR. (1999). *The Use of Antibiotics in Food Producing Animals*. Report of the Joint Expert Advisory Committee on Antibiotic Resistance. Canberra: Commonwealth of Australia Resistance 107–116

[B67] Jones C. M. (2010). *The Effects of Selected Antibiotics on Nitrogen Uptake by Spirodela Punctata.* M.S. thesis, Humboldt State University, Arcata.

[B68] Jones R. L. (2000). Clostridial enterocolitis. *Vet. Clin. N. Am.* 16 471–485 10.1016/s0749-0739(17)30090-1 11219344

[B69] Jukes T. H. (1970). Antibiotics in animal feeds. *N. Engl. J. Med.* 282 49–50 10.1056/NEJM197001012820116 5409472

[B70] JVARM. (2008). *A Report on the Japanese Veterinary Antimicrobial Resistance Monitoring System 2002-2007*. Tokyo: National Veterinary Assay Laboratory, Ministry of Agriculture, Forestry and Fisheries

[B71] Kantas D. Vassilopoulos V. Kyriakis S. C. Saoulidis K. (1998). A dose titration study on the effect of virginiamycin on gilt/sow and piglet performance. *Zentralb. Veterinarmed.* 45 525–533 10.1111/j.1439-0442.1998.tb00856.x 9857830

[B72] Knarreborg A. Lauridsen C. Engberg R. M. Jensen S. K. (2004). Dietary antibiotic growth promoters enhance the bioavailability of alpha-tocopheryl acetate in broilers by altering lipid absorption. *J. Nutr.* 134 1487–1492 15173416 10.1093/jn/134.6.1487

[B73] Kobayashi Y. (2010). Abatement of methane production from ruminants: trends in the manipulation of rumen fermentation Asian-Aust. *J. Anim. Sci*. 23 410–416

[B74] Krausse R. Schubert S. (2010). In-vitro activities of tetracyclines, macrolides, fluoroquinolones and clindamycin against *Mycoplasma hominis* and *Ureaplasma* spp. isolated in Germany over 20 years. *Clin. Microbiol. Infect.* 16 1649–1655 20047607 10.1111/j.1469-0691.2009.03155.x

[B75] Krehbiel C. (2013). The role of new technologies in global food security: Improving animal production efficiency and minimizing impacts. *Anim. Front.* 3 4–7 10.2527/af.2013-0017

[B76] Lallai A. Mura G. Onnis N. (2002). The effects of certain antibiotics on biogas production in the anaerobic digestion of pig waste slurry. *Bioresour. Technol.* 82 205–208 10.1016/S0960-8524(01)00162-6 12003325

[B77] Larsen J. Anderson N. Preshaw A. (2009). Long-acting moxidectin for the control of trichostrongylid infections of sheep in south-eastern Australia. *Aust. Vet. J.* 87 130–137 10.1111/j.1751-0813.2009.00395.x 19335466

[B78] Lindemann M. D. Kornegay E. T. (1989). Folic acid supplementation to diets of gestating-lactating swine over multiple parities. *J. Anim. Sci.* 67 459–464 2703445 10.2527/jas1989.672459x

[B79] Lindemann M. D. Kornegay E. T. Stahly T. S. Cromwell G. L. Easter R. A. Kerr B. J. (1985). The efficacy of salinomycin as a growth promotant for swine from 9 to 97 kg. *J. Anim. Sci.* 61 782–788 4066536 10.2527/jas1985.614782x

[B80] Loftin K. A. Henny C. Adams C. D. Surampali R. Mormile M. R. (2005). Inhibition of microbial metabolism in anaerobic lagoons by selected sulfonamides, tetracyclines, lincomycin, and tylosin tartrate. *Environ. Toxicol. Chem.* 24 782–788 10.1897/04-093R.1 15839550

[B81] Loncaric I. Kubber-Heiss A. Posautz A. Stalder G. L. Hoffmann D. Rosengarten R. (2013). Characterization of methicillin-resistant *Staphylococcus* spp. carrying the mecC gene, isolated from wildlife. *J. Antimicrob. Chemother*. 68 2222–2225 10.1093/jac/dkt186 23674764

[B82] Lovland A. Kaldhusdal M. (2001). Severely impaired production performance in broiler flocks with high incidence of *Clostridium perfringens*-associated hepatitis. *Avian Pathol*. 30 73–81 10.1080/03079450020023230 19184877

[B83] Matthews K. (2001). Antimicrobial drug use and veterinary costs in U.S. livestock production, Agriculture Information Bulletin No. (AIB-766) 761–768

[B84] Manson E. R. (1968). Antibiotics in animal feeds. *Aust. Vet. J.* 44 169–173 10.1111/j.1751-0813.1968.tb09069.x 5689998

[B85] Marshall B. M. Levy S. B. (2011). Food animals and antimicrobials: impacts on human health. *Clin. Microbiol. Rev.* 24 718–733 10.1128/CMR.00002-11 21976606 PMC3194830

[B86] McKellar Q. A. Midgley D. M. Galbraith E. A. Scott E. W. Bradley A. (1992). Clinical and pharmacological properties of ivermectin in rabbits and guinea pigs. *Vet. Rec.* 130 71–73 10.1136/vr.130.4.71 1553807

[B87] Mead P. S. Slutsker L. Dietz V. McCaig L. F. Bresee J. S. Shapiro C. (1999a). Food-related illness and death in the United States. *Emerg. Infect. Dis.* 5 607–625 10.3201/eid0505.990502 10511517 PMC2627714

[B88] Mead P. S. Slutsker L. Griffin P. M. Tauxe R. V. (1999b). Food-related illness and death in the United States reply to Dr. Hedberg. *Emerg. Infect. Dis.* 5 841–842 10.3201/eid0506.990625 10511517 PMC2627714

[B89] Mellata M. (2013). Human and avian extraintestinal pathogenic *Escherichia coli*: infections, zoonotic risks, and antibiotic resistance trends. *Foodborne Pathog. Dis*. 10 916–932 10.1089/fpd.2013.1533 23962019 PMC3865812

[B90] Midtvedt T. (1986). Effects of antimicrobial agents upon the functional part of the intestinal flora. *Scand. J. Infect. Dis.* 49 85–88 3547628

[B91] Moore P. R. Evenson A. (1946). Use of sulfasuxidine, streptothricin, and streptomycin in nutritional studies with the chick. *J. Biol. Chem.* 165 437–441 20276107

[B92] Nagaraja T. G. Taylor M. B. (1987). Susceptibility and resistance of ruminal bacteria to antimicrobial feed additives. *Appl. Environ. Microbiol.* 53 1620–1625 3116929 10.1128/aem.53.7.1620-1625.1987 PMC203920

[B93] NARMS. (2010). *Retail Meat Report – National Antimicrobial Resistance Monitoring System*. Available at: http://www.fda.gov/AnimalVeterinary/SafetyHealth/AntimicrobialResistance/NationalAntimicrobialResistanceMonitoringSystem/default.htm

[B94] Nelson J. M. Chiller T. M. Powers J. H. Angulo F. J. (2007). Fluoroquinolone-resistant *Campylobacter* species and the withdrawal of fluoroquinolones from use in poultry: a public health success story. *Clin. Infect. Dis.* 44 977–980 10.1086/512369 17342653

[B95] Niewold T. A. (2007). The nonantibiotic anti-inflammatory effect of antimicrobial growth promoters, the real mode of action? A hypothesis. *Poult. Sci.* 86 605–609 10.1093/ps/86.4.605 17369528

[B96] Norin K. E. (1997). Influence of antibiotics on some intestinal microflora associated characteristics. *Anaerobe* 3 145–148 10.1006/anae.1997.0091 16887579

[B97] Nunes K. (2008). Hogs raised without antibiotics carry more pathogens. *Food Business News* 12th June.

[B98] Odongo N. E. Bagg R. Vessie G. Dick P. Or-Rashid M. M. Hook S. E. (2007). Long-term effects of feeding monensin on methane production in lactating dairy cows. *J. Dairy Sci.* 90 1781–1788 10.3168/jds.2006-708 17369219

[B99] Partanen K. Siljander-Rasi H. Pentikainen J. Pelkonen S. Fossi M. (2007). Effects of weaning age and formic acid-based feed additives on pigs from weaning to slaughter. *Arch. Anim. Nutr.* 61 336–356 10.1080/17450390701556866 18030917

[B100] Phillips G. (1999). Microbiological aspects of clinical waste. *J. Hosp. Infect.* 41 1–6 10.1016/S0195-6701(99)90029-4 9949957

[B101] Phillips I. (2007). Withdrawal of growth-promoting antibiotics in Europe and its effects in relation to human health. *Int. J. Antimicrob. Agents* 30 101–107 10.1016/j.ijantimicag.2007.02.018 17467959

[B102] Phillips I. Casewell M. Cox T. De Groot B. Friis C. Jones R. (2004a). Antibiotic use in animals. *J. Antimicrob. Chemother.* 53 885 10.1093/jac/dkh149 15028664

[B103] Phillips I. Casewell M. Cox T. De Groot B. Friis C. Jones R. (2004b). Does the use of antibiotics in food animals pose a risk to human health? A critical review of published data. *J. Antimicrob. Chemother.* 53 28–52 10.1093/jac/dkg483 14657094

[B104] Poduval R. D. Kamath R. P. Corpuz M. Norkus E. P. Pitchumoni C. S. (2000). *Clostridium difficile* and vancomycin-resistant enterococcus: the new nosocomial alliance. *Am. J. Gastroenterol.* 95 3513–3515 10.1111/j.1572-0241.2000.03291.x 11151886

[B105] Reports Q. (2012). *China Veterinary Drug Industry 2012 Market Research Report*. Available at: http://www.qyresearchreports.com/report/china-veterinary-drug-industry-2012-market-research-report.htm

[B106] Rodehutscord M. Abel H. J. Friedt W. Wenk C. Flachowsky G. Ahlgrimm H. J. (2002). Consequences of the ban of by-products from terrestrial animals in livestock feeding in Germany and the European Union: alternatives, nutrient and energy cycles, plant production, and economic aspects. *Archiv fur Tierernahrung* 56 67–91 10.1080/00039420214180 12389223

[B107] Rose N. Beaudeau F. Drouin P. Toux J. Y. Rose V. Colin P. (1999). Risk factors for *Salmonella enterica* subsp. enterica contamination in French broiler-chicken flocks at the end of the rearing period. *Prev. Vet. Med.* 39 265–277 10.1016/S0167-5877(99)00002-1 10327442

[B108] Rumpler W. V. Johnson D. E. Bates D. B. (1986). The effect of high dietary cation concentration on methanogenesis by steers fed diets with and without ionophores. *J. Anim. Sci.* 62 1737–1741 3733567 10.2527/jas1986.6261737x

[B109] Russell J. B. Jeraci J. L. (1984). Effect of carbon monoxide on fermentation of fiber, starch, and amino acids by mixed rumen microorganisms *in vitro*. *Appl. Environ. Microbiol.* 48 211–217 6089665 10.1128/aem.48.1.211-217.1984 PMC240371

[B110] Russell S. M. (2003). The effect of air sacculitis on bird weights, uniformity, fecal contamination, processing errors, and populations of *Campylobacter*spp. and *Escherichia coli*. *Poult. Sci.* 82 1326–1331 10.1093/ps/82.8.1326 12943305

[B111] Sarmah A. K. Meyer M. T. Boxall A. B. (2006). A global perspective on the use, sales, exposure pathways, occurrence, fate and effects of veterinary antibiotics (VAs) in the environment. *Chemosphere* 65 725–759 10.1016/j.chemosphere.2006.03.026 16677683

[B112] Scarfe A. D. Lee C.-S O’Bryen P. J. (2011). *Aquaculture Biosecurity: Prevention, Control, and Eradication of Aquatic Animal Disease.* Oxford: Blackwell Publishers

[B113] Schelling G. T. (1984). Monensin mode of action in the rumen. *J. Anim. Sci.* 58 1518–1527 6378867 10.2527/jas1984.5861518x

[B114] Shi J. C. Liao X. D. Wu Y. B. Liang J. B. (2011). Effect of antibiotics on methane arising from anaerobic digestion of pig manure. *Anim. Feed Sci. Technol.* 166–167 457–463 10.1016/j.anifeedsci.2011.04.033

[B115] Soma J. A. Speer V. C. (1975). Effects of pregnant mare serum and chlortetracycline on the reproductive efficiency of sows. *J. Anim. Sci.* 41 100–105 1158786 10.2527/jas1975.411100x

[B116] Sun J. P. Zheng P. Hu B. L. (2009). [Combined effect of antibiotics on anaerobic digestion of piggery wastewater]. Huan jing ke xue = Huanjing kexue / [bian ji, Zhongguo ke xue yuan huan jing ke xue wei yuan hui. *Huan jing ke xue* 30 2619–2624 19927815

[B117] Taylor J. H. Gordon W. S. (1955). Growth-promoting activity for pigs of inactivated penicillin. *Nature* 176 312–313 10.1038/176312a0 13253561

[B118] Tice A. (2001). Outpatient parenteral antimicrobial therapy (OPAT): a global perspective. Introduction. *Chemotherapy* 47(Suppl. 1) 1–4 10.1159/000048562 11096183

[B119] Turnidge J. (2004). Antibiotic use in animals – prejudices, perceptions and realities. *J. Antimicrob. Chemother.* 53 26–27 10.1093/jac/dkg493 14657093

[B120] USFDA. (2009). *Summary Report on Antimicrobials Sold or Distributed for Use in Food-Producing Animals.* Department of Health and Human Services. Available at: http://www.fda.gov/downloads/ForIndustry/UserFees/AnimalDrugUserFeeActADUFA/UCM231851.pdf

[B121] Van Lunen T. A. (2003). Growth performance of pigs fed diets with and without tylosin phosphate supplementation and reared in a biosecure all-in all-out housing system. *Can. Vet. J.* 44 571–576 12892287 PMC340208

[B122] Van Nevel C. Demeyer D. I. (1995). Lipolysis and biohydrogenation of soybean oil in the rumen *in vitro*: inhibition by antimicrobials. *J. Dairy Sci.* 78 2797–2806 10.3168/jds.S0022-0302(95)76910-7 8675762

[B123] Van Nevel C. J. Demeyer D. I. (1977). Effect of monensin on rumen metabolism *in vitro*. *Appl. Environ. Microbiol.* 34 251–257 911159 10.1128/aem.34.3.251-257.1977 PMC242638

[B124] Varga C. Rajic A. McFall M. E. Reid-Smith R. J. Deckert A. E. Checkley S. L. (2009). Associations between reported on-farm antimicrobial use practices and observed antimicrobial resistance in generic fecal *Escherichia coli* isolated from Alberta finishing swine farms. *Prev. Vet. Med.* 88 185–192 10.1016/j.prevetmed.2008.10.002 19041147

[B125] Verkade E. Kluytmans J. (2013). Livestock-associated *Staphylococcus aureus* CC398: animal reservoirs and human infections. *Infect. Genet. Evol*. 21 523–530 10.1016/j.meegid.2013.02.013 23473831

[B126] Vieira A. R. Collignon P. Aarestrup F. M. McEwen S. A. Hendriksen R. S. Hald T. (2011). Association between antimicrobial resistance in *Escherichia coli* isolates from food animals and blood stream isolates from humans in Europe: an ecological study. *Foodborne Pathog. Dis.* 8 1295–1301 10.1089/fpd.2011.0950 21883007

[B127] Vogel G. Nicolet J. Martig J. Tschudi P. Meylan M. (2001). Pneumonia in calves: characterization of the bacterial spectrum and the resistance patterns to antimicrobial drugs. *Schweiz. Arch. Tierheilkd.* 143 341–350 11476040

[B128] Wahlstrom R. C. Terrill S. W. Johnson B. C. (1950). Effect of antibacterial agents on growth of baby pigs fed a “synthetic” diet. *Proc. Soc. Exp. Biol. Med.* 75 710–711 10.3181/00379727-75-18314 14808375

[B129] Weber T. E. Schinckel A. P. Houseknecht K. L. Richert B. T. (2001). Evaluation of conjugated linoleic acid and dietary antibiotics as growth promotants in weanling pigs. *J. Anim. Sci.* 79 2542–2549 11721832 10.2527/2001.79102542x

[B130] Werner G. Coque T. M. Hammerum A. M. Hope R. Hryniewicz W. Johnson A. (2008). Emergence and spread of vancomycin resistance among enterococci in Europe. *Euro Surveill*. 13 pii: 19046 19021959

[B131] Wierup M. (2001). The Swedish experience of the 1986 year ban of antimicrobial growth promoters, with special reference to animal health, disease prevention, productivity, and usage of antimicrobials. *Microb. Drug Resist.* 7 183–190 10.1089/10766290152045066 11442345

[B132] Yano Y. Yokoyama M. Satomi M. Oikawa H. Chen S. S. (2004). Occurrence of *Vibrio vulnificus* in fish and shellfish available from markets in China. *J. Food Prot.* 67 1617–1623 15330524 10.4315/0362-028x-67.8.1617

[B133] Zhang J. J. Wang L. X. Ruan W. K. An J. (2013). Investigation into the prevalence of coccidiosis and maduramycin drug resistance in chickens in China. *Vet. Parasitol.* 191 29–34 10.1016/j.vetpar.2012.07.027 22925822

[B134] Zhou Z. He S. Liu Y. Cao Y. Meng K. Yao B. (2011). Gut microbial status induced by antibiotic growth promoter alters the prebiotic effects of dietary DVAQUA(R) on *Aeromonas hydrophila*-infected tilapia: production, intestinal bacterial community and non-specific immunity. *Vet. Microbiol.* 149 399–405 10.1016/j.vetmic.2010.11.022 21146333

